# 肺癌中*VEGF*基因多态性和临床意义的研究进展

**DOI:** 10.3779/j.issn.1009-3419.2013.08.08

**Published:** 2013-08-20

**Authors:** 明明 胡, 瑛 胡, 宝兰 李

**Affiliations:** 101149 北京，北京市结核病胸部肿瘤研究所，首都医科大学附属北京胸科医院综合科 Department of Medical Oncology, Beijing Chest Hospital Afliated Capital Medical University, Beijing Tuberculosis and Toracic Tumor Research Institute, Beijing 101149, China

1971年Folkman^[1]^第一次提出了血管生成的观点，认为肿瘤的生长、增殖均与血管生成密切相关，并且可以成为肿瘤治疗的靶点。随后的几十年中，无数的肿瘤学家、基础研究科学家、药理学家、制药企业聚集在血管内皮生长因子(vascular endothelial growth factor, VEGF)相关领域，研究VEGF在肿瘤血管生成过程中的分子机制，并以其为药物靶点阻断血管的形成过程，以此抑制肿瘤生长^[2]^。2004年诞生了第一个以血管为靶点的抗VEGF单克隆抗体贝伐单抗，并成功应用于非小细胞肺癌(nonsmall cell lung cancer, NSCLC)、结肠癌和肾癌等实体瘤中。但是，在抗血管生成药物的临床应用过程中，至今还未发现可靠的生物标志物在用药前可以将受益的靶人群筛选出来。正如通过检测表皮生长因子(epidermal growth factor receptor, *EGFR*)突变状态预测应用酪氨酸激酶抑制剂(tyrosine kinase inhibitor, TKI)类药物疗效一样，如果在应用抗血管药物时能做到有的放矢，因材施"治"，将会使患者最大程度上获益。目前研究发现，*VEGF*基因多态性不仅与个体对抗肿瘤药物(包括靶向治疗和化疗)的敏感性有关，还与肿瘤的易感性、生物学行为、预后等方面密切相关，包括乳腺癌、卵巢癌、结直肠癌、前列腺癌等多种实体瘤。但是在肺癌中有关*VEGF*基因多态性的研究还不多，本文就目前肺癌中*VEGF*基因多态性及其临床意义的研究进展做一综述。

## 1 *VEGF*基因简介

1983年，Senger等^[3]^在豚鼠细胞系中发现了一种能导致血管渗出的小分子，命名为血管渗出因子(vascular permeability factor, VPF)；1989年，Ferrara等^[4]^分离和克隆了VEGF，发现其可以诱导内皮细胞的增殖，证明VPF和VEGF是同一种分子。VEGF家族成员包括VEGF-A、VEGF-B、VEGF-C、VEGF-D和血小板生长因子^[5]^。其中，VEGF-A被认为是最重要的一个，通常VEGF指的是VEGF-A。血管生成在肿瘤的增殖、浸润和转移中扮演了重要角色，VEGF无疑是其中最关键的一员。已有研究^[6-8]^证明在多种实体瘤中VEGF mRNA和蛋白过表达，并且与预后相关。*VEGF*基因位于6号染色体6p21.3，包含8个外显子和7个内含子^[9, 10]^。在启动子、5'和3'非翻译区存在多个单核苷酸多态性(single nucleotide polymorphism, SNP)，而且实验室研究证明这些SNP与VEGF在组织中的表达有关^[11]^。目前在肿瘤中研究较多的SNP位点有-2578C > A、-1154G > A、-460T > C、-634G > C、-7C > T、+936C > T。其中-2578C > A、-1154 G > A、-460 T > C在VEGF启动子区，可以影响启动子的活性，从而影响下游的基因转录和翻译^[12]^。-634G > C、-7C > T在5'非翻译区，可以影响与转录因子的亲和力^[11]^。+936C > T在3'非翻译区与VEGF翻译效率相关^[13]^(图 1)。这些SNP位点与肿瘤的易感性、抗肿瘤药物敏感性、预后的关系日益受到关注，并且在肺癌、乳腺癌、胃癌、直结肠癌、卵巢癌等实体瘤中进行了相关研究，表 1将*VEGF*基因多态性与肺癌的遗传易感性、疗效预测和预后之间的关系进行了总结，下文将分别进行叙述。

**1:**
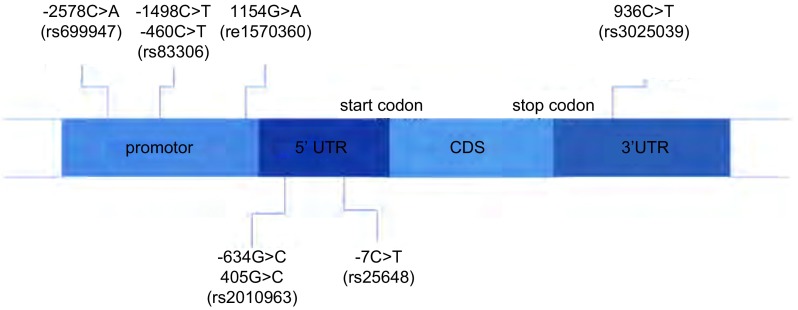
*VEGF*基因结构和SNP位点 Structure of vascular endothelial growth factor (*VEGF*) gene and single nucleotide polymorphism (SNP)

**1:** *VEGF*基因中SNP位点与肺癌易感性、疗效预测和预后之间关系 The relationship between VEGF SNPs and the risk of lung cancer, predictive value to therapy and prognosis

SNP site	Risk of lung cancer	Predictive value	Prognosis
-2578C > A	CA *vs* CC+AA^[16]^↓		
-1154G > A		GG > AG > AA^[22]^	GG *vs* AA+AG^[22]^
-460T > C	TT *vs* CC^[18]^↑		CC+CT *vs* TT^[30]^
	TT *vs* CC+CT^[18]^↑		
-634G > C	CC+CG *vs* GG^[14]^↑		CC+CG *vs* GG^[29]^
	CG *vs* GG; GG *vs* CC ^[16]^↓		
	CC+CG *vs* GG^[15]^↓		
+936C > T	CT+TT *vs* CC^[15, 16]^↓		CC+CT *vs* TT^[29]^
The sign "↑" stands for increasing risk to getting lung caner, while, "↓" means decreasing the risk. In the column of predictive value, compare the response rate of chemotherapy between different genotypes. The comparison of overall survival (OS) are shown in prognosis column. Such as -1154G > A, GG *vs* AA+AG, it means that the patients OS with GG are better than those with AA and AG.			

## 2 *VEGF*基因多态性的临床意义

### 2.1 VEGF基因多态性与遗传易感性

Zhai等^[14]^对*VEGF*的基因多态性与NSCLC易感性进行了研究，分别检测VEGF-460C > T、-634G > C、+936C > T三个位点的多态性，结果显示三个SNP位点在两个人群中的分布未见明显差异，但是分层分析结果显示，-634CC+CG增加了男性腺癌的患病风险(OR=1.40, 95%CI: 1.03-1.87)。单倍型分析显示，五种单倍型CGC、TCC、TGC、CGT、TCT在男性中的分布明显高于对照组，而且与其它四种单倍型相比，CGC型明显降低男性患腺癌的风险(OR=0.76, 95%CI: 0.50-0.98)。在女性中，未发现任何SNP位点和单倍型的与发病风险相关。韩国Lee等^[15]^研究结果显示，-634CC和CG基因型与GG相比，+936CT和T基因型与CC相比，减少了人群中患小细胞肺癌(small cell lung cancer, SCLC)的罹患风险(OR=0.36和0.47，95%CI: 0.17-0.78和0.26-0.85)。单倍型分析，CGT和TCC分别降低和增加患SCLC的风险(OR=0.44和1.63，95%CI: 0.24-0.80和1.14-2.33)。然而，不论NSCLC(包括鳞癌、腺癌、大细胞癌)还是SCLC，单倍型分析(-460T > -634C > +936) TCC和TGT均与发病风险明显相关(OR=0.38和3.94，95%CI: 0.25-0.60和2.00-7.76)。Naykoo等^[16]^的研究支持韩国学者-634CC和CG，+936 CT和TT减少患病风险的的结论(OR=0.07和0.36)，并且还发现-2578CA基因型也可明显降低发病风险(OR=0.08)，单倍型分析CGA和TGA可降低发病风险(OR=0.18, 0.17)。Naik等^[17]^的研究认为-634CG和GG在全部组织学分级、早期、50岁以上肺癌患者中的分布与健康对照有明显性差异。Sun等^[18]^对中国人群中VEGF-460C > T的多态性做了研究，结果显示，T基因型与CC和CT+CC相比，增加发病风险(OR=1.89, 95%CI: 1.17-3.06)，而且TT型在较晚期肺癌中(Ⅲ期和Ⅳ期)的分布明显比早期肺癌多。

### 2.2 *VEGF*基因多态性与生物学行为

检测手术切除的NSCLC患者肿瘤组织和瘤旁组织中四个SNP位点(VEGF-2578C > A、-634G > C、-1154G > A和936C > T)与VEGF表达和微血管密度(microvessel density, MVD)之间的关系时发现，-2578CC、-634GG和-1154AA或GA基因型与VEGF低表达有关，而VEGF高表达与基因型-2578CA、-634GC和-1154GG有关。基因型为-2578CC和-634GG的患者肿瘤组织的MVD比-2578CA+CA和-634CC+GC的患者中计数明显减低。VEGF-1154G > A和936C > T则与肿瘤中VEGF的表达和MVD无关^[19]^。Renner等^[13]^发现936T等位基因与血浆中低VEGF浓度有关。然而Naykoo则认为VEGF的多态性与VEGF表达无关。Maeda等^[20]^检测血液中VEGF基因-460C > T和-634G > C的多态性，以及组织中血管生成相关蛋白HIF、VEGF和Dll4(delta-like ligand 4)的表达，并检测MVD，发现与-460CC基因型相比，TT和TC基因型与MVD和Dll4高表达相关。而VEGF-634G > C SNP与MVD和Dll4表达无关。这一研究结果提示-460C > T或许通过上调Dll4的表达在肿瘤的血管生成过程中发挥作用。故肿瘤在血管生成过程中的不同表现，如微血管数目的不同，血管生成相关蛋白(如VEGF、HIF)的表达差异，或许正是由于基因水平上的差异所造成。

### 2.3 *VEGF*基因多态性与疗效预测

ECOG4599是第一项证明分子靶向药物与化疗药物联合在肺癌治疗中有效的大型Ⅲ期临床研究，该研究奠定了抗血管生成方向在肺癌治疗中的正确性和有效性。基于该项研究，美国FDA批准贝伐单抗联合紫杉醇/卡铂用于一线治疗无脑转移、无出血史的晚期非磷型NSCLC。但是目前为止，没有明确且临床可用的抗血管生成的疗效预测指标，是否可以通过选择有效的预测因子，如同通过检测*EGFR*突变状态预测EGFR-TKI药物的疗效一样，指导选择相对准确的应用抗血管药物的靶向人群，达到精确打击，事半功倍的效果？针对这一问题，科研工作者们在血管内皮生长因子、细胞间粘附分子(intercellular adhesion molecule, ICAM)、胎盘生长因子(placental growth factor, PlGF)、碱性成纤维细胞生长因子(basic fibroblast growth factor, bFGF)等不同的分子标志中希望能够筛选到理想的标志物。

VEGF不仅可以促进新生血管形成而且还能增加血管渗透性，使得间质内流体静压增加，影响了瘤内化疗药物的输送，降低药物的抗肿瘤效果^[21]^。Masago等^[22]^对晚期NSCLC的VEGF-460T > C、-634G > C、+936C > T、-1154G > A、-2587C > A的多态性，并结合化疗疗效，发现-1154G > A的多态性与疾病控制率有关，分别为GG型80.6%，AG型患者68%，AA患者型50%。在结直肠癌、胃癌、卵巢癌中分别有VEGF SNP与化疗联合或不联合贝伐单抗疗效的研究，认为VEGF-460T > C、-634G > C、单倍型AGCGC与疗效有关^[23-27]^。对一线使用舒尼替尼的肾细胞癌患者进行疗效与*VEGF*基因多态性之间关系的研究时发现，-460T > C为T(与CC、CT相比)，-2587C > A为CC(与AA、CA相比)，-634G > C为CC(与GG、GC相比)的患者PFS较短^[28]^。目前研究VEGF SNP与治疗效果之间关系的研究较少，要得到确证性的结论仍有待开展更多的研究。

### 2.4 *VEGF*基因多态性与预后

肿瘤血管常缺乏平滑肌，基底膜上有不规则漏孔，这些漏孔有助于肿瘤细胞进入血液循环，增加远处转移的机会^[29]^。这种结构不完整的血管与VEGF高表达是密切相关的，前文已述*VEGF*基因多态性与VEGF的表达有关系，因此我们关注了*VEGF*基因多态性与患者预后相关的文献。Heist等^[30]^检测外周血细胞基因组中VEGF+936C>T、-460T>C和-634G>C的多态性，发现携带突变型基-634CG+CC比野生型GG明显改善生存(HR=0.70, 95%CI: 0.54-0.90)。+936C>T突变性CT+CC与野生型相比也出现改善趋势(HR=0.73, 95%CI: 0.52-1.03)。分析-634G>C和936C>T两个位点的综合效应时，出现携带突变基因数目越多，生存期越长的趋势，该研究未发现-460T>C与生存有关。而Guan等^[31]^则得到了相反的结论，同样检测VEGF-460T>C、-634G>C和+936C>T三个位点多态性，则发现相反的结论，-460突变型基因即CC+CT，与野生型基因型(TT)相比，可以明显改善患者总生存(HR=0.58, 95%CI: 0.37-0.92)，-634G>C和+936C>T未发现与生存有关。Masago等^[22]^认为-1154G>A、A A和AG基因型是生存的独立预后因子(HR=1.419, 95%CI: 1.309-3.468)。

## 3 展望

在VEGF-VEGFR信号通路中研究最多的是VEGF蛋白的表达与疗效和预后之间的关系，在基因多态性水平上的研究较少，而且现有的研究尚无一致结论，如有的学者认为-1154G>A和936C>T的突变型基因与VEGF低表达有关，有的研究认为-460C>T和-7C>T的突变基因与增加的VEGF mRNA浓度有关^[32, 33]^，甚至同一SNP位点都有互相矛盾的结论，如-936T基因在乳腺癌中认为可以减少发病风险^[34, 35]^，但是在结直肠癌中认为是增加发病风险^[36]^。-634C基因在NSCLC中和前列腺癌中是增加风险的因素^[14, 37]^，在结肠癌中是保护性因素^[38]^，在乳腺癌中与风险无关^[39]^。这些研究结果存在如此多的不一致可能是由于*VEGF*基因中这些SNP位点与其它一些已知或未知的SNP存在连锁的关系，或许单倍型分析比单一的SNP位点分析更有价值，也许是研究群体(民族或瘤种)、样本量(检验效能不足)的因素有关。

VEGF介导的血管生成通路与其他的信号转导通路交织在一起，我们看到的现象或许是这些信号转导通路之间"交互对话"的结果，而不是*VEGF*基因单独作用的结果。但是随着相关研究的开展，人们已经开始关注*VEGF*基因多态性与个体对抗肿瘤药物的敏感性和患者生存率，对肿瘤的易感性、肿瘤生物学行为之间的确存在关联，那么，*VEGF*的基因多态性是否可以作为肺癌临床治疗中更有效的预测和预后指标还有待进行更多、更深入的研究。

## References

[1] Folkman J (1971). Tumor angiogenesis: therapeutic implications. N Engl J Med.

[2] Chou HF, Lin MF, Chen CY (2012). Three-dimensional brain images in preterm children with periventricular leukomalacia. Pediatr Neonatol.

[3] Senger DR, Galli SJ, Dvorak AM (1983). Tumor cells secrete a vascular permeability factor that promotes accumulation of ascites fluid. Science.

[4] Ferrara N, Henzel WJ (1989). Pituitary follicular cells secrete a novel heparin-binding growth factor specific for vascular endothelial cells. Biochem Biophys Res Commun.

[5] Delahanty KM, Myers FE 3rd (2006). Infection control survey. Nursing.

[6] Yuan A, Yu CJ, Chen WJ (2000). Correlation of total VEGF mRNA and protein expression with histologic type, tumor angiogenesis, patient survival and timing of relapse in non-small-cell lung cancer. Int J Cancer.

[7] Yamaguchi T, Bando H, Mori T (2007). Overexpression of soluble vascular endothelial growth factor receptor 1 in colorectal cancer: Association with progression and prognosis. Cancer Sci.

[8] Ferrer FA, Miller LJ, Andrawis RI (1998). Angiogenesis and prostate cancer: in vivo and in vitro expression of angiogenesis factors by prostate cancer cells. Urology.

[9] Brogan IJ, Khan N, Isaac K (1999). Novel polymorphisms in the promoter and 5' UTR regions of the human vascular endothelial growth factor gene. Hum Immunol.

[10] Tischer E, Mitchell R, Hartman T (1991). The human gene for vascular endothelial growth factor. Multiple protein forms are encoded through alternative exon splicing. J Biol Chem.

[11] Watson CJ, Webb NJ, Bottomley MJ (2000). Identification of polymorphisms within the vascular endothelial growth factor (*VEGF*) gene: correlation with variation in VEGF protein production. Cytokine.

[12] Stevens A, Soden J, Brenchley PE (2003). Haplotype analysis of the polymorphic human vascular endothelial growth factor gene promoter. Cancer Res.

[13] Renner W, Kotschan S, Hoffmann C (2000). A common 936 C/T mutation in the gene for vascular endothelial growth factor is associated with vascular endothelial growth factor plasma levels. J Vasc Res.

[14] Zhai R, Liu G, Zhou W (2008). Vascular endothelial growth factor genotypes, haplotypes, gender, and the risk of non-small cell lung cancer. Clin Cancer Res.

[15] Lee SJ, Lee SY, Jeon HS (2005). Vascular endothelial growth factor gene polymorphisms and risk of primary lung cancer. Cancer Epidemiol Biomarkers Prev.

[16] Naykoo NA, Hameed I, Aasif M (2013). Single nucleotide polymorphisms, haplotype association and tumour expression of the vascular endothelial growth factor (*VEGF*) gene with lung carcinoma. Gene.

[17] Naik NA, Bhat IA, Afroze D (2012). Vascular endothelial growth factor A gene (*VEGFA*) polymorphisms and expression of VEGFA gene in lung cancer patients of Kashmir Valley (India). Tumour Biol.

[18] Sun SF, Huang DB, Cao C (2013). Polymorphism of VEGF-460C/T associated with the risk and clinical characteristics of lung cancer in Chinese population. Med Oncol.

[19] Koukourakis MI, Papazoglou D, Giatromanolaki A (2004). *VEGF* gene sequence variation defines *VEGF* gene expression status and angiogenic activity in non-small cell lung cancer. Lung Cancer.

[20] Maeda A, Nakata M, Yasuda K (2013). Influence of vascular endothelial growth factor single nucleotide polymorphisms on non-small cell lung cancer tumor angiogenesis. Oncol Rep.

[21] Jain RK (2002). Tumor angiogenesis and accessibility: role of vascular endothelial growth factor. Semin Oncol.

[22] Masago K, Fujita S, Kim YH (2009). Effect of vascular endothelial growth factor polymorphisms on survival in advanced-stage non-small-cell lung cancer. Cancer Sci.

[23] Oh SY, Kwon HC, Kim SH (2013). The relationship of vascular endothelial growth factor gene polymorphisms and clinical outcome in advanced gastric cancer patients treated with FOLFOX: *VEGF* polymorphism in gastric cancer. BMC Cancer.

[24] Formica V, Palmirotta R, Del Monte G (2011). Predictive value of *VEGF* gene polymorphisms for metastatic colorectal cancer patients receiving first-line treatment including fluorouracil, irinotecan, and bevacizumab. Int J Colorectal Dis.

[25] Etienne-Grimaldi MC, Formento P, Degeorges A (2011). Prospective analysis of the impact of *VEGF*-A gene polymorphisms on the pharmacodynamics of bevacizumab-based therapy in metastatic breast cancer patients. Br J Clin Pharmacol.

[26] Chen MH, Tzeng CH, Chen PM (2011). VEGF-460T→C polymorphism and its association with VEGF expression and outcome to FOLFOX-4 treatment in patients with colorectal carcinoma. Pharmacogenomics J.

[27] Steffensen KD, Waldstrøm M, Brandslund I (2010). The relationship of VEGF polymorphisms with serum VEGF levels and progression-free survival in patients with epithelial ovarian cancer. Gynecol Oncol.

[28] Scartozzi M, Bianconi M, Faloppi L (2013). VEGF and VEGFR polymorphisms affect clinical outcome in advanced renal cell carcinoma patients receiving first-line sunitinib. Br J Cancer.

[29] Zhou QH (2003). Current condition and advances in the target treatment of lung cancer. Zhongguo Fei Ai Za Zhi.

[30] Heist RS, Zhai R, Liu G (2008). VEGF polymorphisms and survival in early-stage non-small-cell lung cancer. J Clin Oncol.

[31] Guan X, Yin M, Wei Q (2010). Genotypes and haplotypes of the *VEGF* gene and survival in locally advanced non-small cell lung cancer patients treated with chemoradiotherapy. BMC Cancer.

[32] Yamamori M, Sakaeda T, Nakamura T (2004). Association of VEGF genotype with mRNA level in colorectal adenocarcinomas. Biochem Biophys Res Commun.

[33] Pander J, Gelderblom H, Guchelaar HJ (2007). Pharmacogenetics of EGFR and VEGF inhibition. Drug Discov Today.

[34] Krippl P, Langsenlehner U, Renner W (2003). A common 936 C/T gene polymorphism of vascular endothelial growth factor is associated with decreased breast cancer risk. Int J Cancer.

[35] Kataoka N, Cai Q, Wen W (2006). Population-based case-control study of *VEGF* gene polymorphisms and breast cancer risk among Chinese women. Cancer Epidemiol Biomarkers Prev.

[36] Bae SJ, Kim JW, Kang H (2008). Gender-specific association between polymorphism of vascular endothelial growth factor (VEGF 936C>T) gene and colon cancer in Korea. Anticancer Res.

[37] Sfar S, Hassen E, Saad H (2006). Association of *VEGF* genetic polymorphisms with prostate carcinoma risk and clinical outcome. Cytokine.

[38] Chae YS, Kim JG, Sohn SK (2008). Association of vascular endothelial growth factor gene polymorphisms with susceptibility and clinicopathologic characteristics of colorectal cancer. J Korean Med Sci.

[39] Schneider BP, Radovich M, Sledge GW (2008). Association of polymorphisms of angiogenesis genes with breast cancer. Breast Cancer Res Treat.

